# Proline–Nitrogen Metabolic Coordination Mediates Cold Priming-Induced Freezing Tolerance in Maize

**DOI:** 10.3390/plants14101415

**Published:** 2025-05-09

**Authors:** Zhijia Gai, Lei Liu, Na Zhang, Jingqi Liu, Lijun Cai, Xu Yang, Ao Zhang, Pengfei Zhang, Junjie Ding, Yifei Zhang

**Affiliations:** 1 Jiamusi Branch, Heilongjiang Academy of Agricultural Sciences, Jiamusi 154007, China; 2 College of Resources and Environment, Jilin Agricultural University, Changchun 130102, China; 3 College of Agronomy, Heilongjiang Bayi Agricultural University, Daqing 163319, China; 4 College of Agronomy, Northeast Agricultural University, Harbin 150030, China

**Keywords:** photosynthesis, proline metabolism, antioxidant enzyme, GDH/ICDH, osmoregulation, ROS

## Abstract

Cold stress critically restricts maize seedling growth in Northeast China, yet the mechanism by which cold priming (CP) enhances cold tolerance through proline–nitrogen metabolic networks remains unclear. This study systematically investigated CP’s synergistic regulation in cold-tolerant (*Heyu27*) and cold-sensitive (*Dunyu213*) maize using a two-phase temperature regime (priming induction/stress response) with physiological and multivariate analyses. CP alleviated cold-induced photosynthetic inhibition while maintaining a higher chlorophyll and photosynthetic rate, though biomass responses showed varietal specificity, with *Heyu27* minimizing growth loss through optimized carbon–nitrogen allocation. Antioxidant enzymes such as superoxide dismutase (SOD), peroxidase (POD), and catalase (CAT) were pre-activated during early stress, effectively scavenging reactive oxygen species (ROS) and reducing malondialdehyde (MDA) accumulation, with *Heyu27* showing superior redox homeostasis. CP enhanced proline accumulation via bidirectional enzyme regulation (upregulating ∆^1^-pyrroline-5-carboxylate synthase/reductase [P5CS/P5CR], inhibiting proline dehydrogenase [ProDH]) and reprogrammed nitrogen metabolism through glutamate dehydrogenase/isocitrate dehydrogenase (GDH/ICDH)-mediated ammonium conversion to glutamate, alleviating nitrogen dysregulation while supplying proline precursors. Principal component analysis revealed divergent strategies: *Heyu27* prioritized proline–antioxidant synergy, whereas *Dunyu213* emphasized photosynthetic adjustments. These findings demonstrate that CP establishes “metabolic memory” through optimized proline–nitrogen coordination, synergistically enhancing osmoregulation, reactive oxygen species (ROS) scavenging, and nitrogen utilization. This study elucidates C_4_-specific cold adaptation mechanisms, advancing cold-resistant breeding and stress-resilient agronomy.

## 1. Introduction

Cold stress commonly occurs during the seedling stage of maize in the Northeast region of China, causing significant damage to the growth and development of the crop. Cold stress (<10 °C) inhibits plant growth and leaf expansion [1]. The productivity of maize (C_4_ crop) is limited by poor photosynthetic performance at low temperatures [2]. Severe cold stress greatly limits maize growth, resulting in substantial yield reductions [3]. Maize yield responses are variety-specific when exposed to cold stress at the seedling stage [4]. This physiological damage is closely associated with the dysregulation of reactive oxygen species (ROS) metabolism [3]. Low temperatures inhibit the activity of the electron transport chain, leading to excessive accumulation of ROS such as superoxide anion (O_2_^−^) and hydrogen peroxide (H_2_O_2_) in the mitochondria and chloroplasts, initiating lipid peroxidation and protein denaturation [5]. Plants maintain ROS homeostasis by activating antioxidant systems such as superoxide dismutase (SOD), peroxidase (POD), and the ascorbate–glutathione cycle, a process that is significantly positively regulated by proline metabolism [6].

During the evolutionary process of coping with low-temperature stress, plants have developed a cold adaptation mechanism centered on osmotic regulation [7]. Proline, as a multifunctional compatible solute, not only maintains water balance by regulating cell osmotic potential but also directly scavenges hydroxyl radicals (·OH) and stabilizes subcellular structures [8]. Studies have shown that proline accumulation induced by low temperature is bidirectionally regulated by ∆^1^-pyrroline-5-carboxylate synthase (P5CS) and proline dehydrogenase (ProDH): cold signals activate P5CS gene expression via an ABA-dependent pathway, promoting the conversion of glutamate to proline; simultaneously, ProDH activity is inhibited to reduce proline degradation [9]. This metabolic reconfiguration allows for a 10–100-fold increase in cytoplasmic proline concentration under cold stress, effectively protecting membrane systems and enzyme activity [10]. Interestingly, proline metabolism is closely linked to nitrogen assimilation. The ammonium assimilation pathway mediated by nitrate reductase (NR) and glutamine synthetase (GS) provides precursor substances for proline synthesis [11]. Under low-temperature stress, the GS/GOGAT cycle is impeded, leading to ammonium (NH_4_^+^) accumulation, which activates P5CS to promote the conversion of glutamate to proline. This rearrangement of nitrogen metabolism alleviates NH_4_^+^ toxicity and enhances osmotic regulation [12]. Recent findings suggest that exogenous spermidine treatment can significantly improve the drought resistance of maize by optimizing the activity of key enzymes in nitrogen metabolism [13], providing a new perspective for enhancing stress resistance through metabolic regulation.

Cold priming, as an agronomic measure to induce resistance, may involve the synergistic regulation of ROS signaling and proline metabolism. Studies have shown that moderate pre-cooling treatment (5–10 °C) can induce H_2_O_2_ signaling through NADPH oxidase, activate the MAPK cascade and CBF transcription factors, and establish a “metabolic memory” [14]. This pretreatment enables plants to activate P5CS gene expression and maintain higher SOD, and CAT activity more rapidly in subsequent stresses [15,16]. Research in wheat has found that cold priming treatment increases the rate of proline accumulation by 2–3 times, along with a 40% increase in GS activity, significantly improving nitrogen use efficiency [17]. The beneficial effects of priming have been documented in several crops, including chickpea [18], sunflower [19], cotton [20], wheat [21], soybean [22], and rice [23]. However, the synergistic regulatory mechanism of the proline metabolism and nitrogen assimilation in maize, a C_4_ crop, has yet to be elucidated, particularly the impact of cold priming on the dynamic balance of proline and the GS/GOGAT cycle requires further investigation.

This study aims to systematically dissect the physiological mechanism by which cold priming regulates cold resistance in maize, hypothesizing the following: (1) cold priming can enhance the cold resistance of maize; (2) different maize varieties (cold-tolerant and cold-sensitive) adopt differentiated strategies to cope with oxidative stress. The mechanism by which cold priming enhances cold resistance in maize through the optimization of the proline–nitrogen metabolic network will be revealed, providing a theoretical basis for the development of stress-resistant cultivation techniques.

## 2. Results

### 2.1. Leaf Dry Weight, Photosynthetic Rate, and Chlorophyll Content

**Figure 1:**
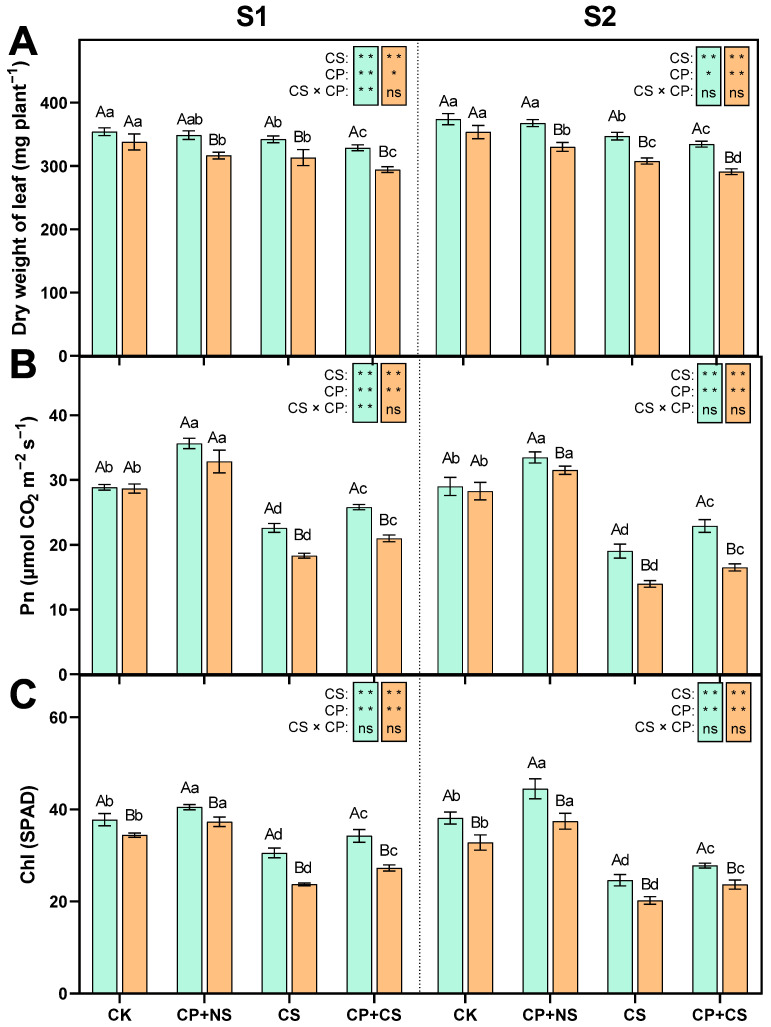
Leaf dry weight (**A**), photosynthetic rate (Pn, **B**), and chlorophyll (SPAD, **C**) in maize as affected by cold priming (CP) and cold stress (CS) after 24 h (S1) and 48 h (S2). CK, normal temperature cultivation plus treatment; CP+NS, cold priming plus no cold stress; CS, normal temperature cultivation plus cold stress; CP+CS, cold priming plus cold stress. The error bar represents the mean ± standard deviation (n = 3). The different capital letters above the numerical column represent statistically significant differences with *p* < 0.05 (Duncan’s range test) between *Heyu27* and *Dunyu213* variety, respectively. The different lowercase letters above the numerical column indicate statistically significant differences among CK, CP+NS, CS, and CP+CS at *p* < 0.05 (Duncan’s range test). The blue bar represents *Heyu27* and the orange bar represents Dunyu 213. The same as follows. **, *, and ns represent significance at 0.01, significance at 0.05, and no significance, respectively. The same as follows.

As shown in Figure 1, compared to CK, the CS and CP+CS treatments significantly reduced the leaf dry weight, photosynthetic rate, and chlorophyll content of both maize varieties at each stage. At the S1 stage, the Pn and Chl contents of both varieties under the CP+CS treatment were 14.15–14.54% and 12.15–14.96% higher than those under the CS treatment, respectively. The CP+NS treatment resulted in an increase in Pn and Chl content compared to CK. Compared to *Dunyu213*, the leaf Pn and Chl contents of *Heyu27* under the CP+CS treatment were significantly increased by 23.05% and 25.59%, respectively. However, the leaf dry weight of both varieties under the CP+CS treatment was 3.90–6.08% lower than that under the CS treatment. Compared to *Dunyu213*, the leaf dry weight of *Heyu27* under the CS and CP+CS treatments was significantly raised by 9.25% and 11.78%, respectively.

At the S2 stage, the Pn and Chl content of both varieties under the CP+CS treatment were 20.27–18.18% and 12.91–17.05% higher, respectively, than those under the CS treatment. Compared to *Dunyu213*, the leaf Pn and Chl contents of *Heyu27* under the CP+CS treatment were significantly increased by 38.70% and 17.53%, respectively. However, the leaf dry weight of both varieties under the CP+CS treatment was 3.62% and 5.49% lower than that under the CS treatment, respectively. Compared to *Dunyu213*, the leaf dry weight of *Heyu27* under the CS and CP+CS treatments was significantly raised by 12.75% and 14.99%, respectively.

### 2.2. ROS Metabolism

**Figure 2:**
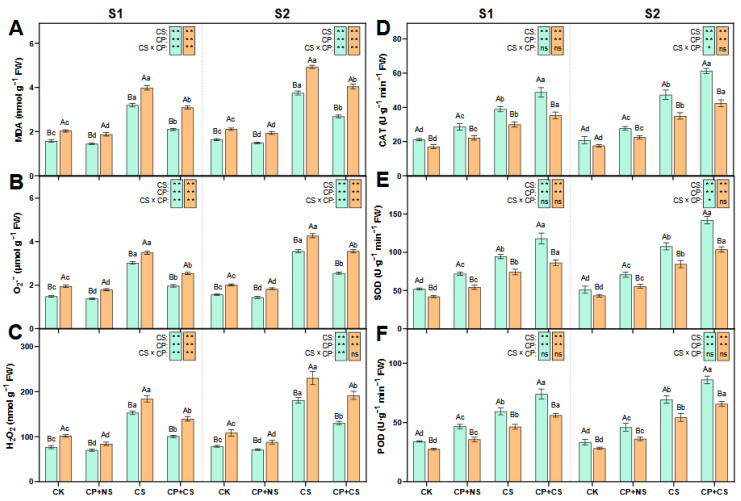
Malondialdehyde (MDA, **A**), superoxide ions (O_2_·^−^, **B**), hydrogen peroxide (H_2_O_2_, **C**), catalase (CAT, **D**), superoxide dismutase (SOD, **E**), and peroxidase (POD, **F**) in maize leaf as affected by cold priming (CP) and cold stress (CS) after 24 h (S1) and 48 h (S2). CK, normal temperature cultivation plus treatment; CP+NS, cold priming plus no cold stress; CS, normal temperature cultivation plus cold stress; CP+CS, cold priming plus cold stress after 24 h (S1) and 48 h (S2). The error bar represents the mean ± standard deviation (n = 3). The different capital letters above the numerical column represent statistically significant differences with *p* < 0.05 (Duncan’s range test) between *Heyu27* and *Dunyu213* variety, respectively. The different lowercase letters above the numerical column indicate statistically significant differences among CK, CP+NS, CS, and CP+CS at *p* < 0.05 (Duncan’s range test).

As indicated in Figure 2, compared to the CK, the CS and CP+CS treatments significantly led to an increase in the content of MDA, H_2_O_2_, and O_2_·^−^, and the activity of SOD, POD, and CAT in both maize varieties at each stage. At the S1 stage, the content of MDA, H_2_O_2_, and O_2_·^−^ in both varieties under the CP+CS treatment was reduced by 34.34–22.43%, 35.06–27.22%, and 34.11–24.17%, respectively, compared to the CS treatment. Compared to *Dunyu213*, the content of MDA, H_2_O_2_, and O_2_·^−^ in *Heyu27* under the CP+CS treatment was significantly reduced by 32.15%, 22.70%, and 27.67%, respectively. Meanwhile, the activity of SOD, POD, and CAT in both varieties under the CP+CS treatment was increased by 24.90–16.46%, 24.64–20.73%, and 25.51–17.56%, respectively, compared to the CS treatment. Compared to *Dunyu213*, the leaf SOD, POD, and CAT activity of *Heyu27* under the CS and CP+CS treatments was significantly raised by 36.70%, 32.03%, and 38.43%, respectively.

At the S2 stage, the content of MDA, H_2_O_2_, and O_2_·^−^ in both varieties under the CP+CS treatment was reduced by 28.41–17.94%, 28.18–16.67%, and 28.37–16.58%, respectively, compared to the CS treatment. Compared to *Dunyu213*, the content of MDA, H_2_O_2_, and O_2_·^−^ in *Heyu27* under the CP+CS treatment was significantly reduced by 33.66%, 32.21%, and 28.30%, respectively. Meanwhile, the activity of SOD, POD, and CAT in both varieties under the CP+CS treatment was reduced by 31.83–22.62%, 23.98–21.07%, and 29.46–21.25%, respectively, when compared to the CS treatment. The leaf SOD, POD, and CAT activity of *Heyu27* under the CS and CP+CS treatments was significantly raised by 37.00%, 30.97%, and 44.99%, respectively, when compared to *Dunyu213*.

### 2.3. Soluble Sugar and Amino Acid Content

**Figure 3:**
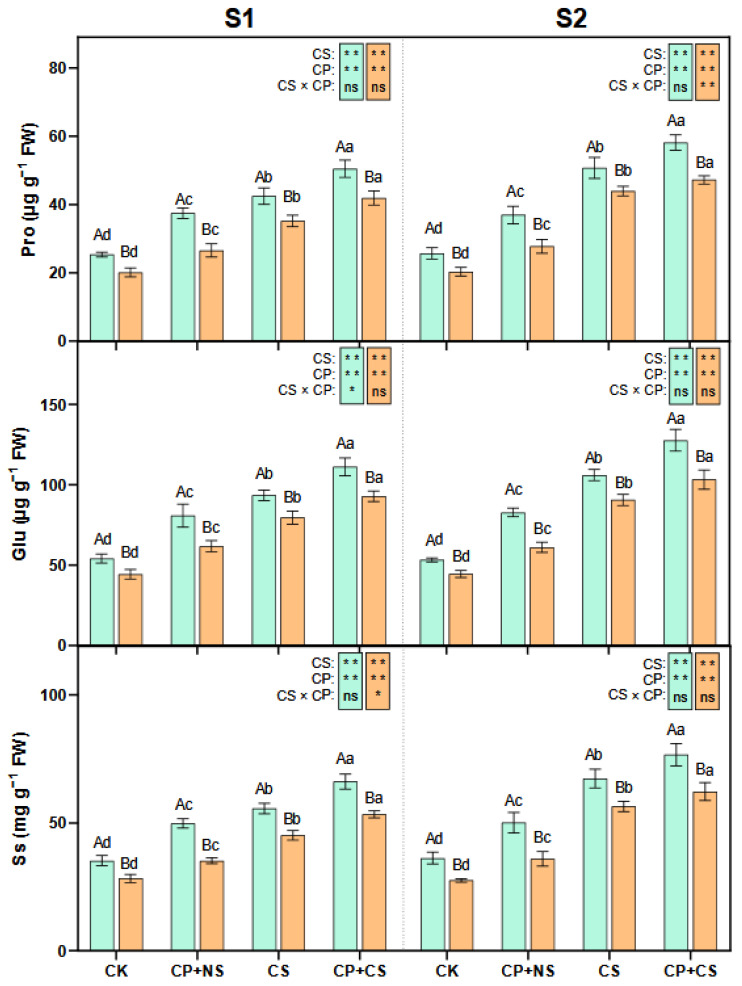
Proline, glutamate, and soluble sugar in maize leaf as affected by cold priming (CP) and cold stress (CS) after 24 h (S1) and 48 h (S2). CK, normal temperature cultivation plus treatment; CP+NS, cold priming plus no cold stress; CS, normal temperature cultivation plus cold stress; CP+CS, cold priming plus cold stress. The error bar represents the mean ± standard deviation (n = 3). The different capital letters above the numerical column represent statistically significant differences with *p* < 0.05 (Duncan’s range test) between *Heyu27* and *Dunyu213* variety, respectively. The different lowercase letters above the numerical column indicate statistically significant differences among CK, CP+NS, CS, and CP+CS at *p* < 0.05 (Duncan’s range test).

As presented in Figure 3, compared to the CK, the CP+NS and CP+CS treatments significantly increased the leaf Pro, Glu, and Ss content of both maize varieties at each stage. At the S1 stage, the Pro, Glu, and Ss content of both varieties under the CP+CS treatment was 18.85–19.02%, 18.92–16.66%, and 18.85–18.16% higher, respectively, than those under the CS treatment. Compared to *Dunyu213*, the leaf Pro, Glu, and Ss content of *Heyu27* under the CP+CS treatment was significantly increased by 20.53%, 19.71%, and 24.04%, respectively.

At the S2 stage, the Pro, Glu, and Ss content of both varieties under the CP+CS treatment was 14.74–7.43%, 20.32–13.97%, and 13.78–10.30% higher, respectively, than those under the CS treatment. Compared to *Dunyu213*, the leaf Pro, Glu, and Ss content of *Heyu27* under the CP+CS treatment was significantly increased by 23.14%, 23.57%, and 23.15%, respectively.

### 2.4. Proline Metabolism Enzymes

**Figure 4:**
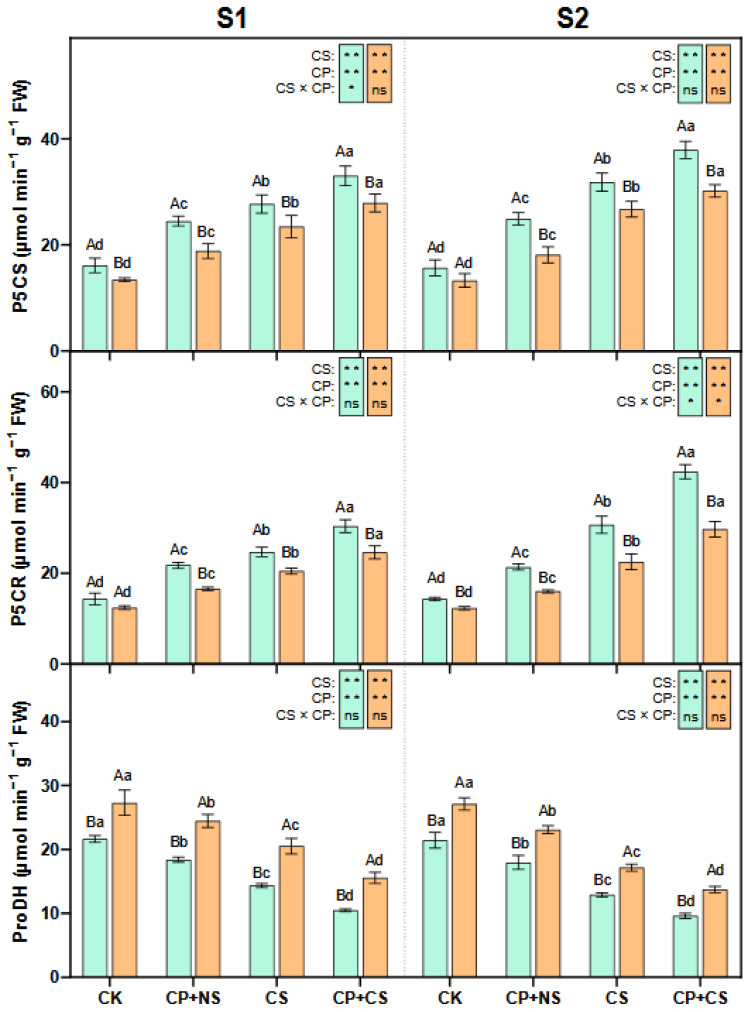
Pyrroline-5-carboxylate synthase (P5CS), pyrroline-5-carboxylate reductase (P5CR), and proline dehydrogenase (ProDH) in maize leaf as affected by cold priming (CP) and cold stress (CS) after 24 h (S1) and 48 h (S2). CK, normal temperature cultivation plus treatment; CP+NS, cold priming plus no cold stress; CS, normal temperature cultivation plus cold stress; CP+CS, cold priming plus cold stress. The error bar represents the mean ± standard deviation (n = 3). The different capital letters above the numerical column represent statistically significant differences with *p* < 0.05 (Duncan’s range test) between *Heyu27* and *Dunyu213* variety, respectively. The different lowercase letters above the numerical column indicate statistically significant differences among CK, CP+NS, CS, and CP+CS at *p* < 0.05 (Duncan’s range test).

As indicated in Figure 4, compared to the CK, the CP+NS and CP+CS treatments significantly enhanced the leaf P5CS and P5CR activities of both maize varieties at each stage, and significantly reduced the ProDH activity. At the S1 stage, the P5CS and P5CR activities of both varieties under the CP+CS treatment were 19.02–18.87% and 22.92–20.06% higher, respectively, than those under the CS treatment. Compared to *Dunyu213*, the leaf P5CS and P5CR activities of *Heyu27* under the CP+CS treatment were significantly increased by 18.20% and 23.45%, respectively. However, the ProDH activity of both varieties under the CP+CS treatment was 27.13% and 24.20% lower, respectively, than that under the CS treatment. The leaf ProDH activity of *Heyu27* under the CP+CS treatment was significantly reduced by 32.83% compared to *Dunyu213*.

At the S2 stage, the P5CS and P5CR activities of both varieties under the CP+CS treatment were 19.07–12.92% and 38.18–32.00% higher, respectively, than those under the CS treatment. Compared with *Dunyu213*, the leaf P5CS and P5CR activities of *Heyu27* under the CP+CS treatment were significantly raised by 25.70% and 42.53%, respectively. However, the ProDH activity of both varieties under the CP+CS treatment was 25.27–19.99% lower than that under the CS treatment. The leaf ProDH activity of *Heyu27* under the CP+CS treatment was significantly reduced by 29.92% compared to *Dunyu213*.

### 2.5. GS-GOGAT Cycle

**Figure 5:**
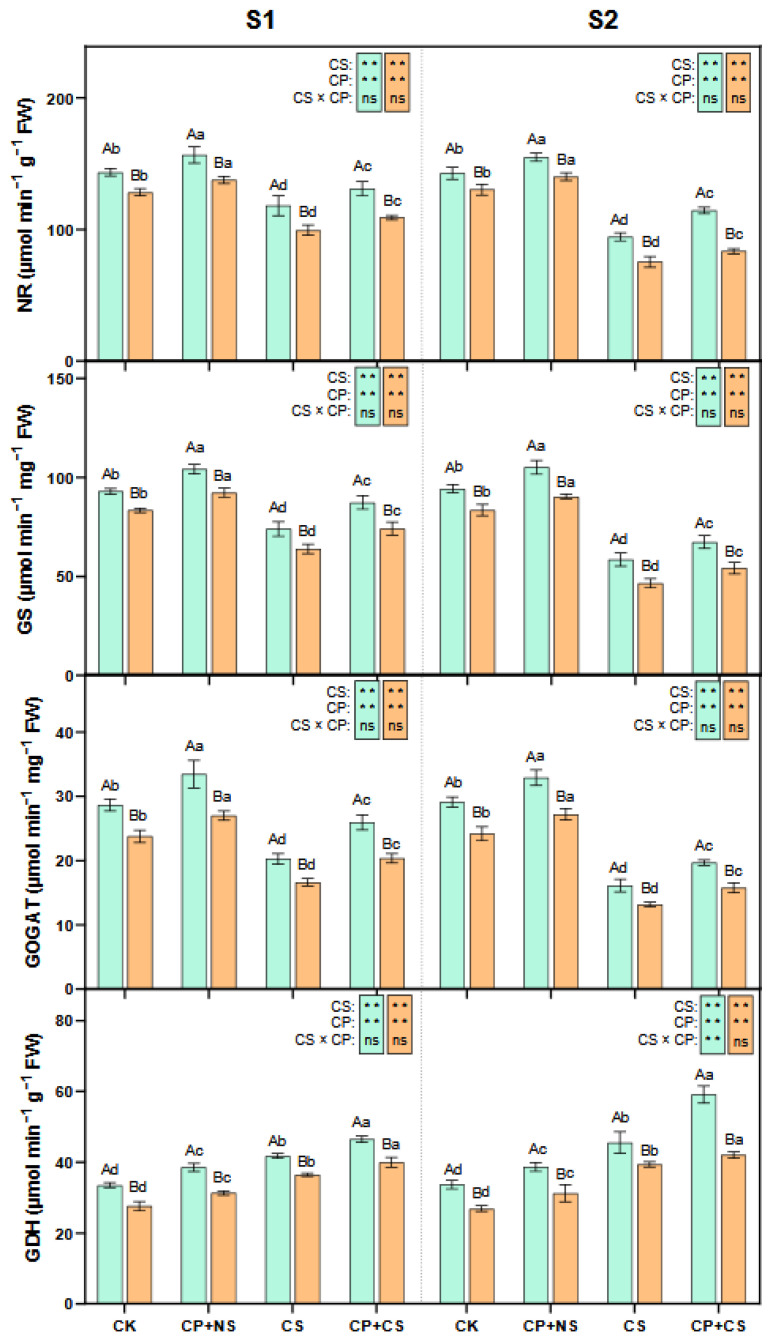
Nitrate reductase (NR), glutamine synthetase (GS), glutamate synthase (GOGAT), and glutamate dehydrogenase in maize leaf as affected by cold priming (CP) and cold stress (CS) after 24 h (S1) and 48 h (S2). CK, normal temperature cultivation plus treatment; CP+NS, cold priming plus no cold stress; CS, normal temperature cultivation plus cold stress; CP+CS, cold priming plus cold stress. The error bar represents the mean ± standard deviation (n = 3). The different capital letters above the numerical column represent statistically significant differences with *p* < 0.05 (Duncan’s range test) between *Heyu27* and *Dunyu213* variety, respectively. The different lowercase letters above the numerical column indicate statistically significant differences among CK, CP+NS, CS, and CP+CS at *p* < 0.05 (Duncan’s range test).

**Figure 6:**
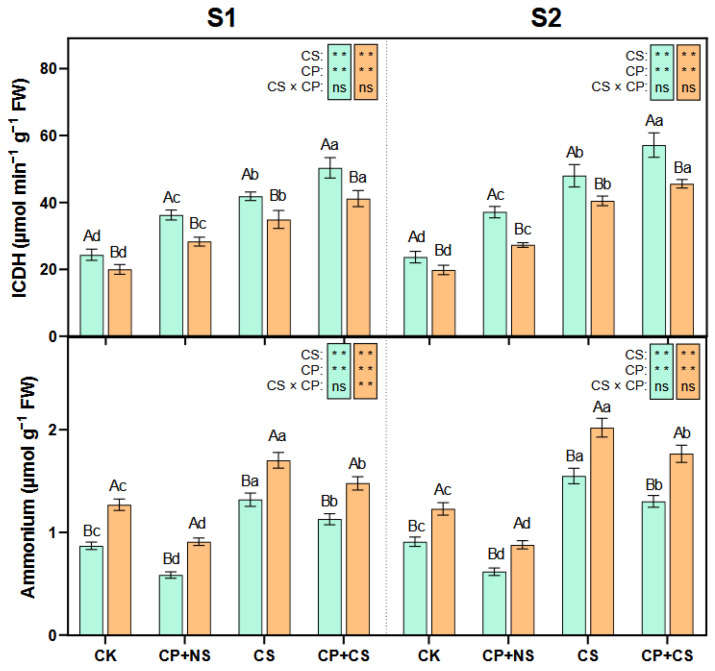
Isocitrate dehydrogenase (ICDH), ammonium in maize leaf as affected by cold priming (CP) and cold stress (CS) after 24 h (S1) and 48 h (S2). CK, normal temperature cultivation plus treatment; CP+NS, cold priming plus no cold stress; CS, normal temperature cultivation plus cold stress; CP+CS, cold priming plus cold stress. The error bar represents the mean ± standard deviation (n = 3). The different capital letters above the numerical column represent statistically significant differences with *p* < 0.05 (Duncan’s range test) between *Heyu27* and *Dunyu213* variety, respectively. The different lowercase letters above the numerical column indicate statistically significant differences among CK, CP+NS, CS, and CP+CS at *p* < 0.05 (Duncan’s range test).

As presented in Figure 5 and Figure 6, compared to the CK, the CS and CP+CS treatments significantly reduced the leaf NR, GS, and GOGAT activities, and significantly increased the GDH, ICDH activities, and ammonium concentration of both maize varieties at each stage. At the S1 stage, the NR, GS, GOGAT, GDH, and ICDH activities of both varieties under the CP+CS treatment were 10.96–9.72%, 17.94–16.13%, 27.91–22.28%, 11.25–9.55%, and 20.20–17.93% higher, respectively, than those under the CS treatment. Compared to *Dunyu213*, the leaf NR, GS, GOGAT, and GDH activities of *Heyu27* under the CP+CS treatment were significantly increased by 20.12%, 17.77%, 27.52%, and 16.41%, respectively. However, the ammonium concentration of both varieties under the CP+CS treatment was 14.39–13.11% lower than that under the CS treatment.

At the S2 stage, the NR, GS, GOGAT, GDH, and ICDH activities of both varieties under the CP+CS treatment were 21.29–10.81%, 15.18–16.53%, 22.31–19.85%, 30.06–6.83%, and 19.07–12.64% higher, respectively, than those under the CS treatment. Compared with *Dunyu213*, the leaf NR, GS, GOGAT, and GDH activities of *Heyu27* under the CP+CS treatment were significantly raised by 37.11%, 24.16%, 24.97%, and 40.82%, respectively. However, the ammonium content for both varieties under the CP+CS treatment was 15.91–12.54% lower than that under the CS treatment.

### 2.6. Multivariate Statistical Analysis

**Figure 7:**
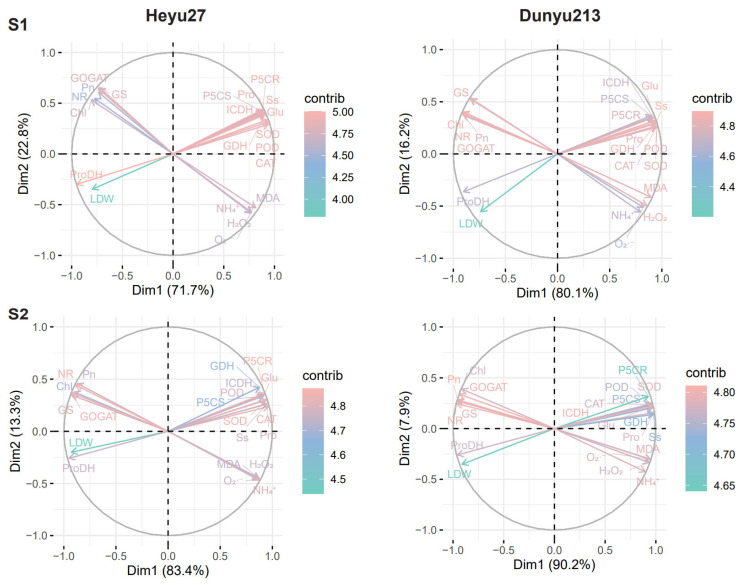
Biplots analysis of principal component among samples of physiological indicators of leaf in maize as affected by cold priming (CP) and cold stress (CS) after 24 h (S1) and 48 h (S2).

Principal component analysis (PCA) was performed on all relevant data to explain the response patterns of maize to the combined stress of cold priming and cold stress (Figure 7). The biplots show a good separation of cold priming and cold stress at both stages. At the S1 stage, PC1 and PC2 of *Heyu27* accounted for 71.7% and 22.8% of the variance, respectively, while those of *Dunyu213* accounted for 80.1% and 16.2%, respectively. At the S2 stage, PC1 and PC2 of *Heyu27* accounted for 83.4% and 13.3% of the variance, respectively, while those of *Dunyu213* accounted for 90.2% and 7.9%, respectively. PC1 clearly distinguished the effect of CS treatment, while PC2 determined the effect of CP treatment. In the S1 stage biplots, the contribution rates of ProDH, SOD, POD, GDH, and ICDH were in the top five for *Heyu27*, while the contribution rates of Chl, Ss, NR, Glu, and POD were in the top five for *Dunyu213*. At the S2 stage, the contribution rates of P5CR, NR, SOD, CAT, and GOGAT were in the top 5 for *Heyu27*, while Pn, NR, ICDH, GOGAT, and GS were in the top five for *Dunyu213*. Therefore, the PCA output suggests that ProDH and P5CR may serve as indicators of cold stress response in *Heyu27*, while Chl and Pn may serve as indicators in *Dunyu213*.

**Figure 8:**
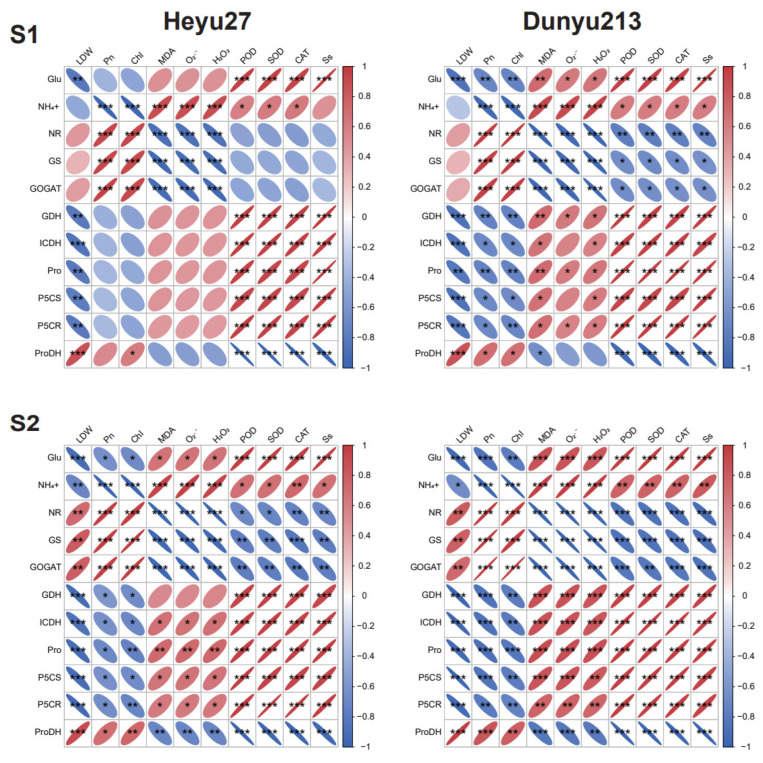
Pearson correlation analysis between proline–nitrogen metabolic and other physiological indicators in maize as affected by cold priming (CP) and cold stress (CS) after 24 h (S1) and 48 h (S2). |r| > 0.8 and *p* < 0.05. LDW, dry wight of leaf; Pn, photosynthetic rate; Chl, chlorophyll; Pro, proline; Glu, glutamate; Ss, soluble sugar; P5CS, pyrroline-5-carboxylate synthase; P5CR, pyrroline-5-carboxylate reductase; ProDH, proline dehydrogenase; NR, nitrate reductase; GS, glutamine synthetase; GOGAT, glutamate synthase; NADHGDH, NADH-dependent glutamate dehydrogenase; NADP-ICDH, NADH-dependent isocitric dehydrogenase; MDA, malondialdehyde; O_2_·^−^, superoxide ions; H_2_O_2_, hydrogen peroxide; CAT, catalase; SOD, superoxide dismutase; and POD, peroxidase. ***, ** and * represent significance at 0.001, significance at 0.01 and significance at 0.05, respectively.

Pearson correlation analysis was performed on the main indicators (Figure 8). The LDW of *Heyu27* and *Dunyu213* was significantly negatively correlated with GDH, ICDH, Pro, P5CS, and P5CR at both stages (*p* < 0.01) and significantly positively correlated with ProDH (*p* < 0.001). At the S1 stage, nitrogen metabolism-related enzymes (NR, GS, and GOGAT) were significantly positively correlated with Pn and Chl, while they were significantly negatively correlated with most ROS (MDA, O_2_·^−^, and H_2_O_2_). ProDH was significantly negatively correlated with POD, SOD, CAT, and Ss. At the S2 stage, nitrogen metabolism-related enzymes (NR, GS, and GOGAT) were significantly positively correlated with Pn and Chl, while they were significantly negatively correlated with most ROS (MDA, O_2_·^−^, and H_2_O_2_) and POD, SOD, CAT, and Ss. Pro, P5CS, and P5CR were significantly negatively correlated with Pn and Chl, while they were significantly positively correlated with MDA, O_2_·^−^, H_2_O_2_, POD, SOD, CAT, and Ss.

## 3. Discussion

### 3.1. The Impact of Cold Priming on Plant Growth and Photosynthetic System

The stability of the plant photosynthetic system is a crucial indicator of its stress resistance [24]. This study, through the combined treatment of cold priming and cold stress, revealed the regulatory characteristics of low-temperature pre-adaptation on the photosynthetic physiology of maize (Figure 1). The results indicated that cold priming significantly mitigated the inhibition of the photosynthetic rate by cold stress and maintained a higher chlorophyll content, a phenomenon consistent with the theory of plants forming adaptive responses through metabolic memory [25]. It is noteworthy that although cold priming treatment improved photosynthetic parameters at both the S1 and S2 stages, its promotional effect on dry leaf weight was variety-specific and closely related to the intensity of stress. From the perspective of the photosynthetic system, cold priming may enhance photosynthetic assimilation capacity through two mechanisms: first, by maintaining the integrity of the thylakoid membrane structure, reducing the damage to photosystem II (PSII) caused by low temperatures [26]; second, by optimizing the activity of key enzymes in the Calvin cycle, such as Rubisco [1,27]. Although C_4_ photosynthesis has a higher potential efficiency of light use than C_3_ photosynthesis [28,29], the productivity of many C_4_ species such as maize, sorghum, and sugarcane is limited by poor photosynthetic performance at low temperature. There was poor photosynthetic performance for C_4_ at low temperatures when compared to C_3_ species [30]. This poor performance may reflect an inherent biochemical limitation, and different steps of C_4_ photosynthesis have been suggested to be the rate-limiting factor at low temperature. Compared to C_3_, there was a photosynthesis constraint imposed by having less Rubisco for the C_4_ plant [27]. For instance, in winter wheat, moderate pre-cooling treatment can reduce photoinhibition by upregulating the expression of genes related to photosynthetic electron transport [15]. In this study, the *Heyu27* variety had a significantly higher photosynthetic rate and chlorophyll content under CP+CS treatment than *Dunyu213*, suggesting a stronger capacity for light capture and conversion efficiency, which may be related to natural variations in chloroplast development regulatory genes. However, the changes in dry leaf weight presented a complex pattern. Although cold priming treatment could mitigate the suppression of photosynthesis by cold stress, the dry leaf weight of the CP+CS group remained lower than that of the CS treatment, which is consistent with observations by Singha et al. [31] under salt-drought cross-stress, and this may be related to energy reallocation strategies [32]. Under cold stress, plants tend to allocate more resources to the synthesis of osmoregulatory substances, such as proline, rather than to biomass accumulation. Similar phenomena have been reported in rice drought resistance research; although drought pretreatment can enhance photosynthetic efficiency, plant dry weight is reduced due to the transfer of carbon sources to soluble sugars [33]. Additionally, the reduction in dry leaf weight of *Heyu27* under cold priming was less than that of *Dunyu213*, indicating that cold-tolerant varieties can optimize carbon–nitrogen allocation efficiency to minimize growth loss while maintaining osmotic balance [34]. Overall, the protective effect of cold priming on the photosynthetic system and the trade-off with dry leaf weight response reflect the resource optimization strategies of plants in stress adaptation. This finding provides a new perspective for elucidating the balance mechanism between stress resistance and productivity.

### 3.2. The Impact of Cold Priming on ROS Metabolism

The steady-state regulation of ROS metabolism is a core mechanism by which plants cope with low-temperature stress [26]. This study found (Figure 2) that cold priming, by pre-activating the antioxidant defense system, significantly reduced the oxidative damage caused by cold stress, with this protective effect being particularly pronounced in the cold-tolerant variety *Heyu27*. In-depth analysis revealed that the regulation of ROS metabolism by cold priming is temporally and spatially specific, involving the dynamic balance of antioxidant enzyme activities and the precise transmission of redox signals [35]. At the S1 stage, the CP+CS treatment significantly increased the activities of SOD, POD, and CAT, thereby effectively scavenging O_2_^−^ and H_2_O_2_ [36]. This phenomenon is consistent with the results of cold acclimation studies in Arabidopsis: pre-cooling treatment can enhance NADPH oxidase-dependent H_2_O_2_ bursts by inducing the MAPK signaling pathway, thereby activating the expression of downstream antioxidant enzyme genes [37]. It is noteworthy that the activity of antioxidant enzymes decreased at the S2 stage, which may be related to energy depletion caused by long-term stress, but the MDA content still remained significantly lower than that of the CS treatment, indicating that the metabolic memory induced by cold priming can delay the process of membrane lipid peroxidation [38].

**Figure 9:**
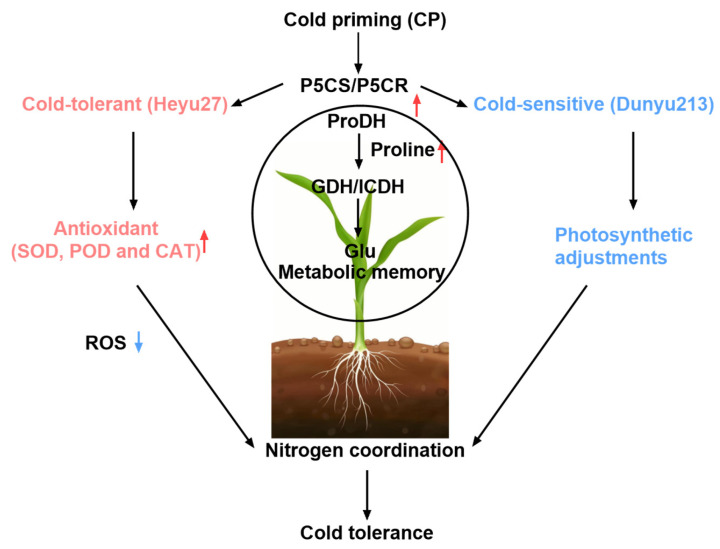
A summarizing schematic diagram of the mechanism of cold priming-induced cold tolerance in maize.

The regulation of ROS signals by cold priming also manifests intervarietal differences. *Heyu27* maintained lower levels of H_2_O_2_ and MDA under CP+CS treatment, indicating a stronger ability to maintain redox homeostasis. This characteristic may arise from two levels: first, a higher baseline activity of antioxidant enzymes, as seen in this study where *Heyu27*’s SOD activity was significantly higher than that of *Dunyu213* at the early stage of stress; second, the direct scavenging of free radicals was through the synergistic action of compatible solutes such as proline [39]. For example, the α-amino group structure of proline can effectively quench singlet oxygen (·OH), and its accumulation level is positively correlated with the efficiency of ROS scavenging [40]. In our study, the leaf SOD, POD, and CAT activity of *Heyu27* under the CS and CP+CS treatments was significantly greater than that with *Dunyu213* (Figure 2). Additionally, PCA showed that SOD and CAT activities are key indicators of *Heyu27*’s response to cold stress, while *Dunyu213* relies more on the adjustment of photosynthetic parameters (Figure 7). This suggests that different varieties may adopt differentiated strategies to cope with oxidative stress: cold-tolerant varieties tend to enhance enzymatic defense systems, while cold-sensitive varieties prioritize the protection of photosynthetic structures (Figure 9). This finding provides important clues for the elucidation of variety-specific cold resistance mechanisms.

### 3.3. Synergistic Regulation of Proline and Nitrogen Metabolism by Cold Priming

The coupling of proline and nitrogen metabolism is a key strategy for plants to adapt to low-temperature stress. As shown in Figure 3 and Figure 4, this study found that cold priming achieved the synergistic optimization of nitrogen reallocation and osmotic balance by synchronously regulating the GS/GOGAT cycle and the proline metabolic pathway [41]. In terms of proline metabolism, cold priming significantly upregulated the activities of P5CS and P5CR while inhibiting ProDH expression. This “diversify and conserve” strategy efficiently converted glutamate into proline, with *Heyu27* demonstrating greater metabolic flexibility in this process. A similar regulatory pattern is seen in wheat cold acclimation research: pre-cooling treatment maintains the sustained activation of the P5CS gene through epigenetic modification, thereby rapidly accumulating proline in subsequent stress [42]. Notably, the accumulation of proline is significantly positively correlated with the activity of GDH, suggesting that it may serve as a nitrogen storage reservoir to mitigate ammonium toxicity. The variety differences in proline accumulation reveals the importance of genetic regulation. The P5CS activity in the cold-tolerant variety *Heyu27* was 18% higher than that in *Dunyu213* (Figure 4), which is related to the increased number of low-temperature response elements (such as MYB binding sites) in the promoter region of the P5CS gene [8]. Compared to Dunyu217, the higher activities of P5CS and P5CR and lower activity of ProDH in *Heyu27* that led to higher proline accumulation in the present study (Figure 4). This bidirectional regulatory mechanism (enhanced synthesis/inhibited degradation) improved the efficiency of proline accumulation in *Heyu27*, significantly enhancing cell membrane stability.

The reprogramming of the nitrogen metabolism is another core response to cold priming. This study found that under cold stress, the GS/GOGAT cycle is obstructed, leading to NH_4_^+^ accumulation, while cold priming activates the GDH pathway to convert excess NH_4_^+^ into glutamate (Figure 5), thereby mitigating ammonium toxicity and providing precursors for proline synthesis (Figure 8). This metabolic conversion contrasts with rice research—C_4_ plants’ unique bundle sheath cells may optimize nitrogen flow through compartmentalized metabolism [43]. Compared to *Dunyu213*, the higher activity of GDH in *Heyu27* under the CP+CS and CS resulted in lower ammonium content in *Heyu27* in the present study (Figure 5 and Figure 6). Cold stress leads to the obstruction of the GS/GOGAT cycle, while cold priming, by inducing the activity of GDH and isocitrate dehydrogenase (ICDH), redirects excess ammonium ions back into the glutamate synthesis pathway. This metabolic shunt not only reduces the toxicity of NH_4_^+^ accumulation to cells but also provides precursor substances for proline synthesis [44]. For instance, in rice salt tolerance research, the increase in GDH activity was proven to be an important compensatory mechanism to mitigate ammonium toxicity [45]. The significant increase in GDH activity in *Heyu27* compared to *Dunyu213* further validates the contribution of nitrogen metabolic flexibility to cold tolerance in varieties. The biological significance of this metabolic synergy lies in the dynamic balance of the proline–nitrogen metabolism network, which allows plants to simultaneously optimize osmoprotection, nitrogen utilization efficiency, and redox homeostasis under low temperature. This mechanism provides a theoretical basis for designing multi-target stress resistance regulatory strategies (Figure 9).

## 4. Materials and Methods

### 4.1. Experimental Materials and Growth Conditions

This trial was performed during the 2023–2024 period at the Jiamusi Branch of the Heilongjiang Academy of Agricultural Sciences experimental site (Latitude 46°48′ N, Longitude 130°22′ E). The experimental materials included the cold-tolerant maize variety *Heyu27* (Registration Number: HeishenYu2016035) and the cold-sensitive variety *Dunyu213* (Registration Number: HeishenYu2016012). A Randomized Block Design (RBD) was employed, with three biological replicates. The potting containers were polyethylene pots with a diameter of 14 cm and a height of 16 cm. The substrate used was the local typical black soil (United States Department of Agriculture soil texture classification: silty clay loam), and the basic physicochemical properties of the plow layer (0–20 cm) soil from soybean field was presented in Table 1. Ten seeds were sown in each pot, with two seedlings per pot ensured after germination. The artificial climate room conditions during the seedling cultivation phase were as follows: day/night temperature of 25 °C/18 °C, relative humidity of 65 ± 8%, light cycle of 14 h light/10 h dark, and soil moisture content maintained at 75 ± 5% using the gravimetric method.

### 4.2. Experimental Design

**Figure 10:**
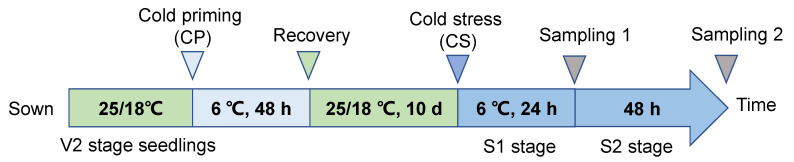
Experimental design.

The formal experiment employed a two-stage temperature treatment system (Figure 10) [46]: the first stage involved cold induction, where seedlings at V2 stage (the second leaf fully expanded) were randomly divided into two groups: the treatment group (CP) was transferred to an artificial climate chamber for low-temperature induction at 6 ± 0.5 °C (light cycle 14 h/10 h, photosynthetically active radiation 300 μmol·m^−2^·s^−1^, relative humidity 65 ± 3%), while the control group (CK) was maintained at a suitable temperature of 20 ± 1 °C, for a duration of 48 h. The second stage involved cold stress treatment, after 10 days of recovery cultivation (day/night temperature 25 °C/18 °C), the CP and CK groups were each divided into two subgroups: the CS subgroup underwent gradient cooling to a target temperature of 6 ± 0.5 °C, while the control group remained at 20 ± 1 °C. This resulted in four treatment combinations: CK (constant temperature cultivation + constant temperature treatment), CP+NS (cold induction + no cold stress), CS (constant temperature cultivation + cold stress), and CP+CS (cold induction + cold stress), with three biological replicates for each treatment (n = 12 plants/replicate).

Leaf samples were collected 24 h (S1, osmoregulation stage) and 48 h (S2, steady-state response stage) after cold stress treatment. During sampling, a pre-cooled stainless steel punch (diameter 1 cm) was used to collect leaf disk samples, avoiding the midrib, which were then flash-frozen in liquid nitrogen and stored at −80 °C for later use. Physiological index measurements were conducted on fresh samples immediately after sampling, with three technical replicates set for each time point.

### 4.3. Measurement of Photosynthetic Parameters and Chlorophyll Content

Net photosynthetic rate (Pn) was measured using a CI-340 portable photosynthesis system (CID Bio-Science, Camas, WA, USA) between 9:00 and 11:00 AM, with a CO_2_ concentration set at 400 μmol·mol^−1^ and light intensity at 1200 μmol·m^−2^·s^−1^. The relative chlorophyll content (SPAD value) was determined using a SPAD-502 chlorophyll meter (Konica Minolta, Tokyo, Japan), with three sites selected per leaf, and averaging the readings.

### 4.4. Measurement of Key Enzyme Activities in Nitrogen Metabolism

The activity of nitrate reductase (NR) was determined in accordance with naphthylamine colorimetric method and the absorbance was measured at 540 nm [47]. The activities of glutamine synthetase (GS), glutamate synthase (GOGAT), and NADH-glutamate dehydrogenase (NADH-GDH) were determined by employing the spectrophotometry method at 540 nm [48], 540 nm [49], and 340 nm [50], respectively. The activity of isocitrate dehydrogenase (ICDH) was determined by monitoring the isocitrate-dependent rate of NADH^+^ reduction at 340 nm [45]. The ammonium content was determined by fluorimetry with HPLC-system, and the excitation wavelength and emission wavelength were 410 nm and 470 nm, respectively [45].

### 4.5. Measurement of ROS Contents and Key Enzyme Activities in ROS Scavenging

The O_2_⋅^−^ production rate was determined using the hydroxylamine oxidation method and the OD value was determined at 530 nm [51]. The malonaldehyde (MDA) content was evaluated using the 2-thiobarbituric acid (TBA) method, and the MDA content was measured at 450, 532, and 600 nm [52]. Following the method of Pompeu et al., the samples were homogenized in 0.1% (*w*/*v*) TCA for the hydrogen peroxide (H_2_O_2_) determination with potassium iodide, and the absorbance which was read at 390 nm [53].

Superoxide dismutase (SOD) activity was assayed by the nitrogen blue tetrazolium (NBT) method at 560 nm, peroxidase (POD) was measured by the oxidation rate of guaiacol at 470 nm and catalase (CAT) was determined by a decrease in absorbance per minute at 240 nm [54].

### 4.6. Determination of Amino Acids and Soluble Sugar

Leaves were pulverized in cold 75% ethanol. After 12 min, the homogenates were centrifuged, and the supernatant was collected for the determination of the free amino acid content. The content of glutamate (Glu) was quantified by using fluorimetry with the HPLC-system [55]. Proline (Pro) was determined using the ninhydrin colorimetric method and the absorbance was measured at 520 nm [56]. The soluble sugar (Ss) concentration was determined with the anthrone colorimetric method and the absorbance was assayed at 620 nm [57].

### 4.7. Measurement of Proline Metablism Enzymes

The activity of ∆^1^-pyrroline-5-carboxylate reductase (P5CR) was determined by a ultraviolet spectrophotometry method and the activity was determined by a decrease in absorbance per minute at 340 nm [58]. The activity of pyrroline-5-carboxylate synthetase (P5CS) was determined by a spectrophotometer method at 340 nm [59]. The activity of proline dehydrogenase (ProDH) was assayed with the spectrophotometry method and the absorbance subsequent to the reaction was read at 450 nm [59].

### 4.8. Statistical Analysis

SPSS 22.0 (IBM, Armonk, NY, USA) was used for two-way analysis of variance (Two-way ANOVA), with cold start treatment and stress duration as fixed factors and variety as a random factor. Significant differences between groups were tested using Duncan’s range test (*p* < 0.05), and data are presented as mean ± standard deviation. Principal Component Analysis (PCA) and heatmap visualization were performed using GraphPad Prism 9.0.

## 5. Conclusions

This study systematically elucidates the physiological mechanisms by which cold priming enhances the cold resistance of maize. Cold priming pre-activates the antioxidant defense system, significantly reducing the accumulation of ROS and membrane lipid peroxidation induced by cold stress, with the cold-tolerant variety *Heyu27* demonstrating a stronger ability to regulate redox. Simultaneously, cold priming induces the synergistic reprogramming of proline metabolism and nitrogen assimilation: on the one hand, it promotes proline accumulation by upregulating P5CS/P5CR activity and inhibiting ProDH expression; on the other hand, it optimizes ammonium assimilation efficiency through the GDH pathway, mitigating nitrogen metabolic disorders. It is noteworthy that the trade-off between the improvement of photosynthetic parameters and dry leaf weight response reveals the complexity of regulating resistance and growth balance. These findings not only clarify the uniqueness of the cold adaptation mechanism in maize but also provide key theoretical support for the selection of cold-tolerant varieties and the development of stress-resistant cultivation technologies.

## Figures and Tables

**Table 1:** Soil properties (0–20 cm) before the experiment.

Total N (g/kg)	Available N (mg/kg)	Total P (g/kg)	Available P (mg/kg)	Total K (g/kg)	Available K (mg/kg)	Organic Matter (g/kg)	pH
2.87 ± 0.11	148.65 ± 4.65	1.65 ± 0.09	32.21 ± 3.23	29.53 ± 2.78	132.43 ± 4.21	32.43 ± 2.91	7.08 ± 0.05

Note: total N (Total nitrogen); available nitrogen (Available N); total phosphorus (Total P); available phosphorus (Available P); total potassium (Total K); available potassium (Available K).

## Data Availability

The data presented in this study are available on request from the corresponding authors. The data are not publicly available due to intellectual property rights.
